# Numerical Modeling Using Immersed Boundary-Lattice Boltzmann Method and Experiments for Particle Manipulation under Standing Surface Acoustic Waves

**DOI:** 10.3390/mi14020366

**Published:** 2023-01-31

**Authors:** Fatima Alshehhi, Waqas Waheed, Abdulla Al-Ali, Eiyad Abu-Nada, Anas Alazzam

**Affiliations:** 1 Mechanical Engineering Department, Khalifa University, Abu Dhabi P.O. Box 127788, United Arab Emirates; 2 System on Chip Lab, Khalifa University, Abu Dhabi P.O. Box 127788, United Arab Emirates

**Keywords:** microfluidics, Lattice Boltzmann, immersed boundary, acoustofluidics, standing surface acoustic wave, microparticle

## Abstract

In this work, we employed the Immersed Boundary-Lattice Boltzmann Method (IB-LBM) to simulate the motion of a microparticle in a microchannel under the influence of a standing surface acoustic wave (SSAW). To capture the response of the target microparticle in a straight channel under the effect of the SSAW, in-house code was built in C language. The SSAW creates pressure nodes and anti-nodes inside the microchannel. Here, the target particle was forced to traverse toward the pressure node. A mapping mechanism was developed to accurately apply the physical acoustic force field in the numerical simulation. First, benchmarking studies were conducted to compare the numerical results in the IB-LBM with the available analytical, numerical, and experimental results. Next, several parametric studies were carried out in which the particle types, sizes, compressibility coefficients, and densities were varied. When the SSAW is applied, the microparticles (with a positive acoustic contrast factor) move toward the pressure node locations during their motion in the microchannel. Hence, their steady-state locations are controlled by adjusting the pressure nodes to the desired locations, such as the centerline or near the microchannel sidewalls. Moreover, the geometric parameters, such as radius, density, and compressibility of the particles affect their transient response, and the particles ultimately settle at the pressure nodes. To validate the numerical work, a microfluidic device was fabricated in-house in the cleanroom using lithographic techniques. Experiments were performed, and the target particle was moved either to the centerline or sidewalls of the channel, depending on the location of the pressure node. The steady-state placements obtained in the computational model and experiments exhibit excellent agreement and are reported.

## 1. Introduction

Advances in microfluidics and microfabrication technologies over the last two decades have allowed researchers to develop microfluidics-based techniques and devices for precise manipulation and regulation of fluids and analytes suspended in the working fluids. Consequently, these devices can handle a variety of samples, including reagents, particles, cells, subcellular components, and other biological bodies. These devices, in particular, hold great importance in numerous industrial and academic fields related to the food industry [1], chemical analyses [2], environment screening [3], and health/clinical sciences [4]. The microfluidics-based devices leverage the advancements in microfabrication technologies and employ micron-sized conduits called microchannels to handle tiny fluid volumes (even up to pico- and femtoliters). An important application of microfluidic devices is to develop lab-on-a-Chip (LOC) microdevices, where the goal is to integrate most of the laboratory processes into a single platform. In addition, these microfluidics-based LOC devices offer various advantages, including low costs, greater sensitivity and selectivity, shorter processing times [5], lower sample volume requirements, and higher accuracy and repeatability [6]. Because of these positive attributes, many LOC devices are increasingly being developed for multiple applications.

Based on their operation, the manipulation methods in microfluidic LOC devices can be classified into active and passive techniques. The active methods employ single or multiple external force fields to manipulate the analytes in the microchannel. These may include magnetic [7], acoustic [8], optical [9], electric [10], and mechanical [11] forces that have been harnessed efficiently to control the movement of target samples. On the other hand, passive methods do not use external forces for their operation. Instead, these methods manipulate the particle depending on the hydrodynamic forces and the geometrical features for particle manipulation. Some common passive methods are inertial focusing, hydrophoresis, deterministic lateral displacement, pinched flow fractionation, and filtration [12]. The active techniques often handle the samples with relatively more control and can be easily tuned in real-time. However, their flow rates are limited because the applied force fields must compete and surpass the hydrodynamic drag to steer the samples effectively. Consequently, the Reynolds number $\left(Re\right)$—a ratio of inertial to viscous forces—in such devices is quite low. In contrast, the passive techniques involve simpler operations, are generally robust, and entail relatively large flow rates; but, in comparison to active techniques, they lack precision in the manipulation of the analytes. Among the active LOC devices, the acoustic-based (also termed acoustofluidic) platforms have received particular attention due to their simplicity, accuracy, efficiency, non-invasive nature, and label-free operations [13]. In the acoustofluidic platforms, a surface acoustic wave (SAW) field generates an acoustic radiation force that affects the particles and causes their motion [14]. A SAW is a mechanical wave that travels parallel to the surface of an elastic material, and its amplitude decreases exponentially with depth into the material [13]. The generated force moves the fluid medium and particles if its acoustic properties are different from the surrounding medium. When SAWs are overlapped, standing surface acoustic waves (SSAW) are generated. The SSAW causes a pressure difference in the channel and creates a pressure node (where acoustic radiation force is zero) inside the microchannel. The location of the pressure node depends on the SAW wavelength and the width of the microchannel. The position of a microparticle in the microchannel changes with time. The particle’s response under the SSAW depends on various parameters. These include the particle and fluid densities, size of the particle, speed of sound, and the amount of energy density of the standing acoustic wave. The primary acoustic radiation force acting on a particle whose radius r is much less than the wavelength of the sound wave in an inviscid fluid is shown in Equations (1) and (2) [15].

(1) $$F_{A}=-\frac{2\pi V_{p}E_{ac}}{\lambda }\Phi \left(\beta ,\rho \right)\sin(\frac{4\pi y_{p}}{\lambda })$$

(2) $$\Phi (\beta ,\rho )=\frac{5\rho_{p}-2\rho_{f}}{2\rho_{p}+\rho_{f}}-\frac{\beta_{p}}{\beta_{f}}$$

where $V_{p}$ is the volume of the particle, $E_{ac}$ is the acoustic energy density which is related to the applied voltage, such that $E_{ac}=aV^{2}$. (with a being the constant of proportionality), β and ρ represent the compressibility and density. The term Φ is the acoustic contrast factor that dictates the particle motion toward the pressure node or antinode of the acoustic wave. If Φ is positive, the particle tends to move towards the pressure nodes, whereas it advances towards the anti-nodes for a negative Φ value. Most particles have a positive value of Φ; thus, they tend to move toward the pressure nodes. The limitation of this method is that SSAW is less effective near the sides of the channel due to the propagation properties of SAW. This can decrease the separation efficiency if the particles are placed near the sides. However, if it is necessary to place the particles on the channel sides, sheath fluid is required to move them to the SSAW-high effective area [16].

Over the years, numerical simulations have emerged as crucial tools in understanding the underlying mechanism and the complicated interplay of the multiple physical processes in microfluidics platforms. The systematic simulations allow accurate forecasting of the performance of these platforms. Thus, they not only aid in decreasing the processing times but also are influential in decreasing the overall cost of the system by eliminating the costly trial-and-error exercises in the experiments. To this end, various numerical techniques have been introduced to solve complicated multiphysics-based systems. Among these techniques, continuum-based computational fluid dynamics (CFD) methods such as the finite difference method, finite volume method, and finite element method have been developed throughout the years to solve fluid dynamics problems. These methods solve the macroscopic variables of interest (such as pressure and velocity) by discretizing the Navier–Stokes Equations (NSEs), which describe the flow of an incompressible working fluid in the microchannel as:

(3) ∇·u=0

(4) $$\rho \left[\frac{\partial u}{\partial t}+\left(u\;\cdot \nabla \right)u\right]=-\nabla P+\mu \nabla^{2}u+F_{ext}$$

where ρ represents the density, u is the velocity, and μ denotes the viscosity of the working fluid. The term ∇P represents the pressure gradient, and $F_{ext}$ is a body force applied per unit volume on the fluid. In the current work, $F_{ext}$ is the force generated by the acoustic field. The motion of the particles suspended in the working fluid is described by Newton’s second law.

(5) $$m\frac{du}{dt}=F_{total}$$

where $F_{total}$ represents the summation of all forces acting on the target microparticle. Solving the non-linear NSEs, however, is a challenging task and involves memory, time, and resource-intensive processes. In addition, with the increasing complexity of sample preparation techniques, there exists a high difference in the length and time scales of the solutes and solvents, which makes it challenging for these CFD methods to completely predict the working of physical systems, especially in the case of a large suspension of sample particles, and their mutual interaction cannot be ignored. To deal with these problems, several particle-based mesoscopic simulation methods, such as molecular dynamics (MD), smoothed particle hydrodynamics, Brownian dynamics [17], and dissipative particle dynamics [18,19] have been developed. The most detailed among these techniques is the MD which calculates the interactions on atomic/molecular levels. However, it cannot be used beyond nano-scaled systems as the number of particles representing the larger physical systems becomes unrealistically huge, thus increasing the time and computations [20].

The Lattice Boltzmann Method (LBM) [21] is a particle-based alternate CFD numerical technique that simulates complex geometries and fluid flows. The LBM is a mesoscopic method introduced to solve the problem of lattice gas automata (LGA), which was developed first by Hardy, Pomeau, and De Pazzis in 1973 [22]. The main disadvantages of LGA were noise, non-Galilean invariance, and high values of numerical viscosities. The correct NSEs using hexagonal lattice were found by Frisch, Hasslacher, and Pomeau in 1986 [23]. In 1988, McNamara and Zanetti introduced the LBM and solved many issues in LGA [24]. Due to the capability of the LBM to deal with complex geometries, it is suitable for modeling flow in porous media. The LBM is based on a discrete model and mesoscopic kinetic equations. It has several advantages, making it a promising technique for numerically solving fluid and heat flow problems. The incompressible continuity equation can be satisfied in the LBM without solving the non-linear NSEs [21]. The pressure values are obtained directly from the state equation using the LBM, whereas in CFD, the Poisson equation needs to be solved after each iteration to find the pressure value. Additionally, its algorithm is more straightforward and can solve cases with complicated boundary conditions. Furthermore, the LBM is easy to apply for multi-phase and multi-component flows without tracking the interfaces between the different phases. However, the LBM requires more computer memory than the Navier–Stokes method, which is not a big issue these days.

Various investigations have been performed previously to understand the behavior of microparticles within acoustofluidic devices. Most of these works are based only on experiments because modeling the acoustofluidic phenomenon, in general, is comparatively more difficult than performing the experiments. Nevertheless, some excellent efforts by researchers exist in developing reliable numerical models to better understand the fundamental physics of the acoustofluidic processes and support the development of SAW-based devices without the need to conduct lengthy experimental works [23,25]. Gantner et al. [26] performed numerical simulations of SAWs based on a mathematical model that illustrated the coupling of the piezoelectric substrate and elastomechanical phenomena. Barnkob et al. [27] developed a new method of calculating the local pressure amplitude in a microchannel caused by bulk acoustic waves. The method depends on the tracking of individual polystyrene (PS) particles in the microchannel. The motion of the particles was recorded and fitted to a theoretical curve to calculate the pressure amplitude. The pressure field and the acoustic potential were numerically calculated for the entire microchannel. Muller et al. [28] established a numerical model to study the motion of particles exposed to acoustic radiation force and acoustic streaming force inside rectangular-shaped microchannels based on the comprehensive perturbation method. Finite element software (COMSOL Multiphysics) was used to develop a model to simulate the particle acoustophoretic motion inside the microchannel. To build the model, the first-order acoustic field of a standing surface wave was determined among the microchannel by solving the linearized compressional Navier–Stokes equation, continuity equation, and heat equation. Then, the calculated first-order acoustic field was used to calculate the streaming flow and the acoustic radiation forces. Then, the time-dependent trajectories of particles were calculated. Using the same numerical approach, Nama et al. [29] successfully modeled the acoustophoretic motion of particles inside a microchannel made of polydimethylsiloxane (PDMS) and flowing within an isentropic compressible liquid exposed to standing acoustic waves. The new model showed significantly different acoustic fields than those observed in bulk acoustic wave devices. Moreover, no acoustic streaming rolling inside the viscous boundary was observed. Mao et al. [30] established a two-dimensional SSAW acoustofluidic model based on the acoustophoresis theory. The model considers the effects of boundary vibrations, channel materials, and channel dimensions on acoustic waves’ propagation. The model was validated experimentally for acoustofluidic device design and optimization. Another experimentally validated numerical model was developed by Guo et al. [31]. The work illustrated the relationship between the acoustic vibrations, standing acoustic field, and streaming in a microfluidic by modeling and experimental validation. Three-dimensional acoustic tweezers have been presented experimentally and numerically. The tweezers used standing surface acoustic waves to generate 3D trapping nodes that captured and manipulated particles. The standing acoustic wave moved particles in-plane, whereas the amplitude of acoustic vibrations controlled particle motion along an orthogonal plane. Another work based on the finite element method to model acoustophoretic devices using COMSOL was reported by Skov and Bruus [32]. The modeled microchannel consisted of a straight water-filled microchannel with an elastic wall of either hard wall pyrex or soft wall PDMS placed on top of a piezoelectric transducer that created standing waves. The results showed that the full model used to simulate the pyrex wall produced fairly good results. On the other hand, the soft wall model used for PDMS simulation resulted in a poor approximation. The experiment concluded that the modeling of acoustofluidic devices should be performed in full models to take into account all effects related to the elastic walls of the microchannel. Bach and Bruus [33] extended and implemented the theory of pressure acoustics in the numerical finite element method using COMSOL by taking into consideration the viscous effects of a thin boundary layer and derived a slip-velocity boundary condition for steady acoustic streaming. Using this approach, the new method improved the computational efficiency of the fluidic domain. A meshless analytical model has been proposed by O’Rorke [34]. The model is used for rapid calculation of the acoustic pressure field. The model was developed using MATLAB software (MATLAB R2017a, The MathWorks Inc., Natick, MA, USA), and another sister model was developed using COMSOL for benchmark comparison and validation. An experimental model was also developed for further validation of the method using traveling and standing acoustic waves. In a recent study, Ni et al. [35] discussed the acoustic behaviors of PDMS based on standing surface acoustic waves while building numerical models. The study compared three PDMS definition assumptions: solid elastic material, non-flow fluid, and lossy wall. In another recent study, Skov et al. [36] presented a 3D numerical simulation taking into account the electromechanical fields of the piezoelectric acoustofluidic device, the acoustic displacement field in the attached elastic material, the acoustic fields inside the microchannel, and the resulting acoustic radiation force and streaming force acting on the suspended particles. A prediction of the acoustic resonances and the acoustophoretic response of the suspended particles in 3D was compared to an experimental result. The developed model correctly predicted the existence and position of the observed in-plane streaming-flow rolls.

In the current work, we developed a numerical model complemented by in-house experiments to investigate the motion of a microparticle in a straight microchannel under the effect of an external SSAW force. The numerical method employed in the current work is the IB-LBM which provides a bidirectional coupling between the suspension fluid flow and the microparticle immersed in it. Moreover, the current approach is efficient since, unlike conventional continuum-based methods, it does not involve mesh destruction and regeneration to capture the particle’s motion. The model is first validated against various popular benchmark cases, and an excellent agreement is obtained. Next, the developed model is used to simulate an important operation in the LOC devices called “focusing” of microparticles in which the microparticles flowing randomly in the microchannel are deflected to form a single stream under the action of the applied SSAW force. The location where the microparticles are focused depends on the position of the SSAW pressure node. In the numerical model, the pressure node is changed to a different location (such as the centerline or sides of the microchannel), and the successful deflection of the particle towards the node is demonstrated. Moreover, various parametric studies are carried out in which the effect of multiple parameters on the trajectory of the microparticle is investigated. Finally, a microfluidic platform comprising a piezoelectric wafer bonded with a PDMS microchannel is fabricated to complement the numerical results. Experiments are designed to replicate the numerical conditions, and the results demonstrate that both are in close agreement.

## 2. Mathematical Modeling

### 2.1. Model for Fluid, Particle, and Their Interaction

**Figure 1:**
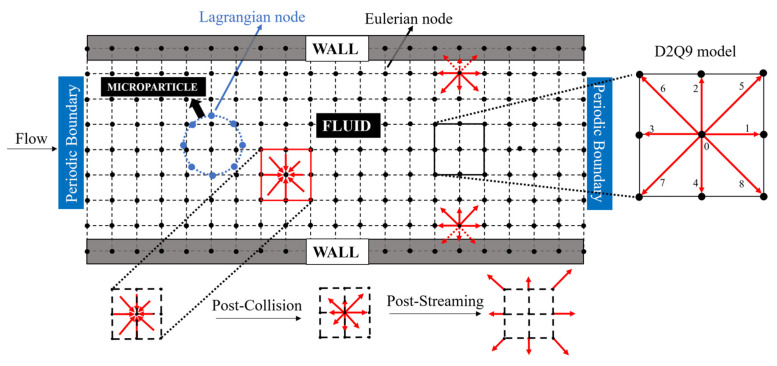
Schematics of the IB-LBM computational domain based on the D2Q9 model and the boundary conditions.

In this section, all the essential details of the numerical technique employed in the current work are described. Figure 1 represents a schematic diagram of the computational framework along with the boundary conditions. As mentioned above, the LBM is based on a discrete model and mesoscopic kinetic equations. It describes the fluid flow by fictitious particles that occupy discrete lattice sites called ‘nodes’ and move with discrete velocities to their neighboring sites in prescribed directions based on the model used [37]. For example, a $D_{N}Q_{M}$ represents an n-dimensional Eulerian grid with M velocity directions. In the current work, a D2Q9 model is utilized, which means it simulates 2-D fluid flow with nine discrete velocities $e_{i}$ (i=0,1,…,8). The fluid flow is computed by the Lattice Boltzmann Equation (LBE), a special case of the continuous Boltzmann Equation in which space, time, and velocity are discretized as shown in Equation (6) [37]:

(6) $$f_{i}\left(X+e_{i}\Delta t,t+\Delta t\right)-f_{i}\left(X,t\right)=\Omega_{i}\left(X,t\right)+F_{i}\left(X,t\right)\Delta t,\;\;\left(i=0,1,\ldots 8\right).\;$$

where $f_{i}$ is the ‘discrete distribution function’ that represents the probability of locating a particle on a node X at time t. The particle moves from one node to another with a velocity $e_{i}$. $\Omega_{i}$. is the collision operator, and $F_{i}$ is the external force. At each time step, the particles move along these links, collide with other particles, and exchange energy and momentum through two main processes in the LBM: streaming and collision. In the streaming phase, the particle moves from one lattice node to an adjacent node. In the collision phase, the particle collides with another particle from an adjoining node [37]. The macroscopic density ρ and momentum density ρu are related to the distribution function $f_{i}$ as shown in the following equations [21]:

(7) $$\rho =\overset{M}{\underset{i=1}{\sum }}f_{i}$$

(8) $$\rho u=\overset{M}{\underset{i=1}{\sum }}f_{i}e_{i}$$

The simplest approximation of the collision operator was introduced by Bhatnagar, Gross, and Krook in their famous model called the BGK model [21]:

(9) $$\Omega_{i}\left(X,t\right)=-\frac{1}{\tau }\left(f_{i}\left(X,t\right)-f_{i}^{eq}\left(X,t\right)\right)\;\;\;\;\;\;\;\;\left(i=0,1,\ldots 8\right)$$

where τ represents the relaxation time, and $f^{eq}$ is the equilibrium distribution function. For the D2Q9 model, the equilibrium distribution function is given as [21]:

(10) $$f_{i}^{eq}=\rho w_{i}\left[1+\frac{e_{i\;}\cdot u}{C_{s}^{2}}+\frac{{\left(e_{i\;}\cdot u\right)}^{2}}{2C_{s}^{4}}-\frac{u^{2}}{2C_{s}^{2}}\right]$$

where $C_{s}$ is the sound speed with a numerical value of 1/√3. The lattice weight coefficients are [37]:

(11) $$w_{i}=\left[\begin{array}{c} 4/9;\;i=0\\ 1/9;\;i=1,2,3,4\\ 1/36;\;i=5,6,7,8 \end{array}\right]$$

Finally, the viscosity in the LBM is linked to the relaxation time by:

(12) $$\nu =C_{s}^{2}\left(\tau -0.5\Delta t\right)$$

The microparticle is modeled as a closed membrane comprising connected lagrangian nodes. These lagrangian nodes, unlike fluid particles, do not follow the lattice nodes and can take any position in the computational domain. The interaction of the particle membrane and the flowing fluid is implemented via the IB-LBM. Peskin introduced the Immersed boundary method (IBM) in 1971 to simulate flow patterns around heart valves [38]. In 1998, Eggleton and Popel first utilized the LBM to simulate the flow of blood cells [39]. Later, Feng and Michaelides successfully combined the LBM with the IBM for the first time to simulate rigid particle motion [40]. The boundary force on the membrane (of microparticle) is a function of the deformation of the membrane and is computed by the constitutive relations (such as Hooke’s Law or any other constitutive law). The computed force is then dispersed to the Euler nodes of the lattice by using a specific Dirac delta function. Furthermore, the immersed microparticle moves in the fluid in such a way that the zero-slip boundary condition on the interface between the membrane and fluid is satisfied. This means each lagrangian node of the membrane moves at the same velocity as that of the fluid in its vicinity.

Following Peskin’s work, the body force is spread to the local lattice nodes via the relation [41]:

(13) $$F\left(x,t\right)=\sum_{i}^{N}F_{b}\;\delta \;\left(X-x_{i}\left(t\right)\right)$$

where the term X represents the location of the lattice (Euler) node in the flow field, $x_{i}$ is the ith (Lagrangian) node, $F_{b}$ represents the force on the lagrangian nodes, N defines the number of boundary nodes, and $\delta \left(X-x_{i}\left(t\right)\right)$ is the Dirac function to interpolate $F_{b}$ from the Lagrangian nodes of the membrane to the Eulerian (lattice) node of the flow field. Following Peskin, the Dirac delta function used in the current work is of the form:

(14) $$\delta \left(x\right)=\left\{\begin{array}{c} 1-\left|x\right|\;\;\;\;\;\;\;\;\;\;\;\;\;\;\;\;\;0\leq \left|x\right|\leq 1\\ \;0\;\;\;\;\;\;\;\;\;\;\;\;\;\;\;\;\;\;\;\;\;\;1\leq \left|x\right| \end{array}\right.$$

The process of force spreading affects the local fluid velocity; hence, the Shan–Chen velocity shift Scheme [42] is employed to represent the shift in the fluid velocity $\left(u\right)$ due to the force $F_{b}$. A new equilibrium velocity $\left(u^{eq}\right)$ is computed as:

(15) $$u^{eq}=u+\frac{\tau }{\rho }F_{b}\Delta t$$

According to the Shan and Chen scheme [40], this velocity should replace the velocity term in Equation (10) to compute the equilibrium distribution function $f_{i}^{eq}\left(x\right)$ to translate the Force effect successfully on the fluid flow field. This change in the fluid flow field is then transferred to the moving membrane’s nodes via. interpolation, and the velocity of the Lagrangian point, $\dot{x_{i}}$, is calculated as [41]:

(16) $${\dot{x}}_{i}\left(t+1\right)=\underset{X}{\sum }u\left(X,t+1\right)\delta \;\left(X-x_{i}\left(t\right)\right)$$

It should be noted that the same Dirac delta function that was introduced for the force spreading is used in the velocity interpolation step. In the last step, the immersed membrane’s position is computed via the Euler method, and the results are saved for plotting/post-processing. For more details about the IB-LBM and the interaction of the fluid and immersed objects, the reader is referred to refs [43,44].

In addition to the Immersed boundary methods, a variety of other boundary conditions are also employed in the current work. The vertical boundaries of the microchannel are assigned a periodic boundary condition. As a result, the microchannel is modeled with an infinite length in the x direction. Two stationary walls are assigned to the top and bottom of the microchannel. These two walls consist of LB particles with zero velocities. A bounce-back condition is set to the stationary walls to ensure that the fluid does not penetrate the stationary walls or exit the fluid domain.

### 2.2. Parameter Mapping from Physical to LBM Domain

The LBM model works in a reduced system of units different from the actual physical units. Thus, it is very important to map the physical parameters onto the LBM domain to visualize the physical phenomena correctly. This is achieved by non-dimensionalizing the governing term and matching the non-dimensional terms (such as Re) in both domains [45]. The governing equations in the current case are converted to their non-dimensional forms by introducing the terms:

$$u^{*}=\frac{u}{U_{f}},\;\nabla^{*}=H\nabla ,\;t^{*}=\frac{tU_{f}}{H},P^{*}=\frac{P}{\rho U_{f}^{2}},\;\rho^{*}=\frac{\rho_{p}}{\rho_{f}},D^{*}=\frac{D}{H},\;\lambda^{*}=\frac{\lambda }{H}\;\&\;y^{*}=\frac{y}{H}$$

Here, $U_{f}$ is the characteristic velocity of the flow, which is the maximum velocity of the fluid flow in the microchannel, and H defines the characteristic length (here, microchannel width). The non-dimensional equations can be stated as [37]:

(17) $$\nabla^{*}\cdot u^{*}=0$$

(18) $$\left[\frac{\partial u^{*}}{\partial t^{*}}+\left(u^{*}\;\cdot \nabla^{*}\right)u^{*}\right]=-\nabla^{*}P^{*}+\frac{\nu }{U_{f}H}\nabla^{*}{}^{2}u^{*}+F_{ext}^{*}$$

As can be seen, the above equations yield a critical dimensionless quantity called the Reynolds number (Re = $\frac{U_{f}H}{\nu })$. Therefore, matching Re is the starting point to model the physical phenomenon in the IB-LBM. Next, the simulation parameters are set, and the conversion factors for parameters such as length, density, and viscosity are set as: $C.F._{l}=\frac{W_{\left(phy\right)}}{Ny_{\left(LBM\right)}\;}$, $C.F._{\rho }=\frac{\rho_{\left(phy\right)}}{\rho_{\left(LBM\right)}\;}$, and $C.F._{\nu }=\frac{\nu_{\left(phy\right)}}{\nu_{\left(LBM\right)}\;}$, respectively. The subscript phy is used for physical while LBM denotes the numerical value of the parameter. The details of the parameters and their conversion factors are given in Table 1:

Using the conversion factors mentioned in Table 1, all other conversion factors for all different derived quantities were computed. For instance, the conversion factor for the velocity was calculated as $C.F._{velocity}=\frac{C.F._{l}}{C.F._{t}}$. Similarly, the conversion factor for the force was calculated as $C.F._{Force}$= ${\left[C.F._{velocity}\right]}^{2}{\left[C.F._{l}\right]}^{2}\left[C.F._{\rho }\right]$.

**Figure 2:**
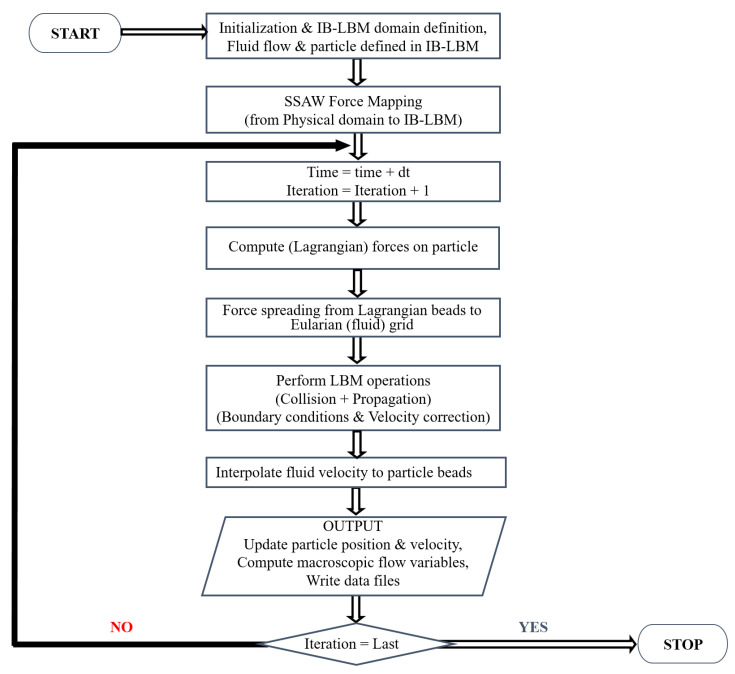
The flow chart diagram depicting the algorithm implemented in the current work.

The essential computations and the algorithm that followed to establish the interaction between the fluid and the immersed particle are shown in the flow chart in Figure 2.

## 3. Verification and Validation

In the first verification study, we developed our LBM code to simulate the Poiseuille flow and match the results with the analytical solution of the Hagen–Poiseuille flow profile [45]. The streamwise component of velocity ($u_{x}$) in the Hagen–Poiseuille profile inside a microchannel separated by plates by a distance H is analytically stated as [45]:

(19) $$u_{x}\left(y\right)=-\frac{dP}{dx}\frac{H^{2}}{2\mu }\;\;\left(1-\frac{y^{2}}{H^{2}}\right)$$

**Figure 3:**
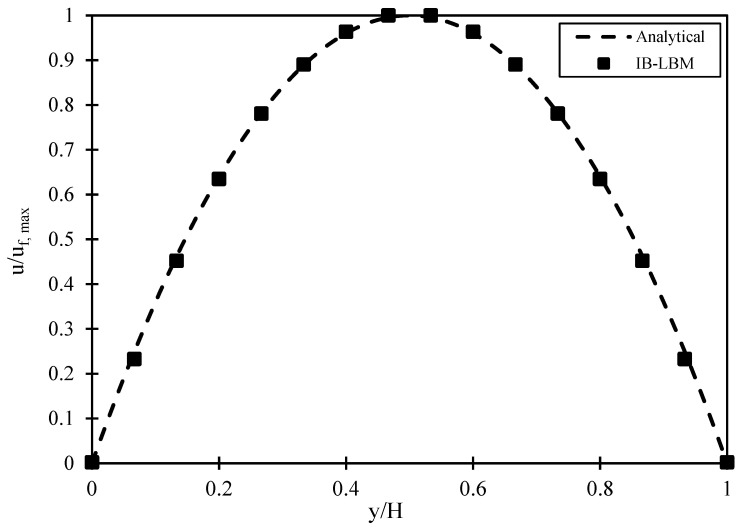
Velocity profile (normalized) of Poiseuille flow in the microchannel and its comparison with the Hagen–Poiseuille analytical solution.

Here, $\frac{dP}{dx}$ is the pressure gradient that generates the flow in the x-direction. The D2Q9 lattice arrangement represents the fluid domain of 40 × 40 units with a fixed layer outside each horizontal boundary, representing fixed horizontal plates of the microchannel. A bounce-back boundary condition is applied at each fixed plate, which mainly implies that an incoming particle towards the solid boundary bounces back into the flow domain [46]. For instance, according to this scheme, the unknown functions, namely $f_{2}$, $f_{5}$, and $f_{6}$ for the bottom plate after bounce-back are computed as $f_{2}=f_{4},\;f_{5}=f_{7},f_{6}=f_{8}$ where $f_{3},\;f_{6}$, and $f_{7}$ are already known from the streaming step. A periodic boundary condition is applied to the vertical boundaries, which means that any particle leaving the domain from the right boundary will be introduced back into the domain from the left boundary. Figure 3 shows the normalized parabolic velocity profile generated by the in-house code and its validation against the analytical solution. It can be seen that the velocity is maximum at the centerline of the microchannel with a value of $u=u_{f,max},\;$ and zero at the walls. The numerical results match the analytical results and demonstrate a correct implementation of the zero-slip boundary condition.

In the second benchmarking study, the built code was developed further to simulate the velocity distribution in a lid-driven cavity case. In this study, the fluid is confined to four walls (Top, Bottom, Left, and Right), and the fluid flow in response to the motion of the Top wall in the horizontal direction is investigated. The details of the boundary conditions applied to each wall are mentioned in ref [21]. The Top wall moves from left to right, and the boundary conditions assigned to this boundary are:

(20) $$\rho_{N}=\frac{1}{1+V_{y,N}}[f_{8}+f_{0}+f_{2}+2(f_{1}+f_{5}+f_{4})\;$$

(21) $$f_{3}=f_{1}-\frac{2}{3}\rho_{N}V_{y,N}$$

(22) $$f_{6}=f_{4}+\frac{1}{2}\left(f_{0}-f_{2}\right)-\frac{1}{6}\rho_{N}V_{y,N}-\frac{1}{2}\rho_{N}V_{x,N}$$

(23) $$f_{7}=f_{5}+\frac{1}{2}\left(f_{2}-f_{0}\right)+\frac{1}{2}\rho_{N}V_{x,N}-\frac{1}{6}\rho_{N}V_{y,N}$$

A bounce-back boundary condition is assigned for the stationary bottom wall, as explained in Equations (24)–(26).

(24) $$f_{1}=f_{3}$$

(25) $$f_{4}=f_{6}+\frac{1}{2}\left(f_{2}-f_{0}\right)$$

(26) $$f_{5}=f_{7}+\frac{1}{2}\left(f_{0}-f_{2}\right)$$

Since both vertical boundaries are also stationary, the bounce-back boundary condition is used for both vertical walls. Therefore, for the right (vertical) boundary, the following boundary conditions are used:

(27) $$f_{2}=f_{0}$$

(28) $$f_{6}=f_{4}+\frac{1}{2}\left(f_{1}-f_{3}\right)$$

(29) $$f_{5}=f_{7}+\frac{1}{2}\left(f_{3}-f_{1}\right)$$

In the same manner, the left (vertical) wall is assigned the following boundary conditions

(30) $$f_{0}=f_{2}$$

(31) $$f_{4}=f_{6}+\frac{1}{2}\left(f_{3}-f_{1}\right)$$

(32) $$f_{7}=f_{5}+\frac{1}{2}\left(f_{1}-f_{3}\right)$$

**Figure 4:**
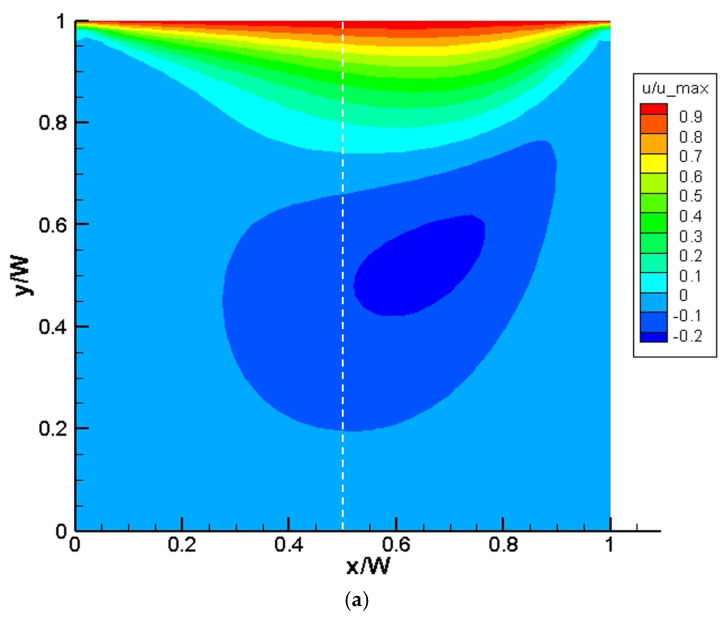

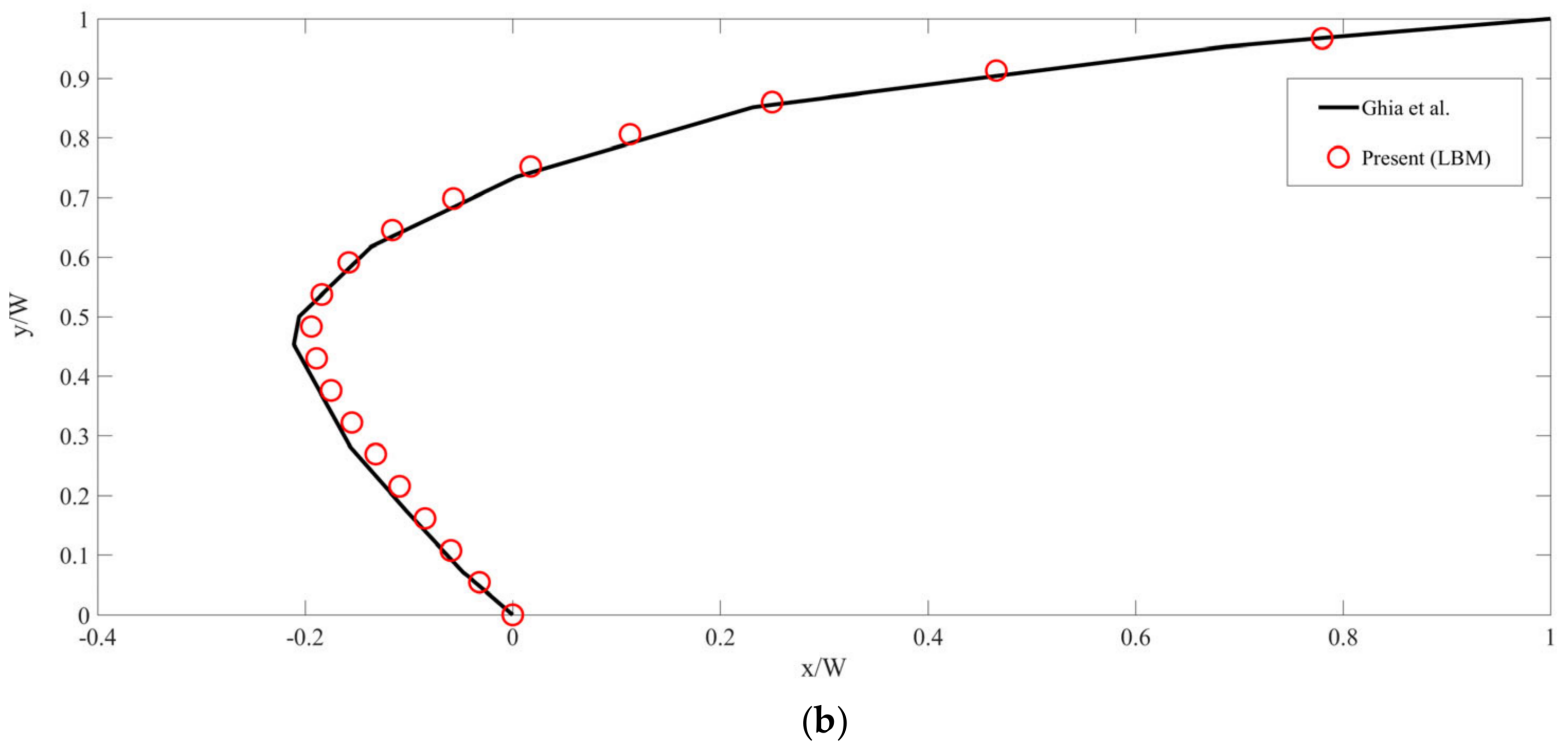
(**a**) Contour plot of velocity distribution for lid-driven cavity case. The vertical dashed line passes through the geometric center of the domain. (**b**) The horizontal velocity component along the dashed line obtained in the LBM model and its comparison with results reported by Ghia et al. [47].

For the Top wall, $V_{y,N}=0\;$ since it moves only in the horizontal direction. A numerical domain with dimensions $L_{x}\times L_{y}$ = 50×50 is defined, and the top wall is displaced from left to right in the positive x direction such that Re = 100 where the Re is based on the velocity of the Top wall. The results obtained in the model are validated with the results reported by Ghia et al. [47], as shown in Figure 4a,b. Figure 4a displays the normalized velocity in the computational domain. Figure 4b shows the horizontal velocity component obtained in the simulation along the vertical line passing through the geometrical center of the computational domain and compares it with that reported by Ghia et al. [47]. Again, a good agreement between the numerical results and literature data is established.

**Figure 5:**
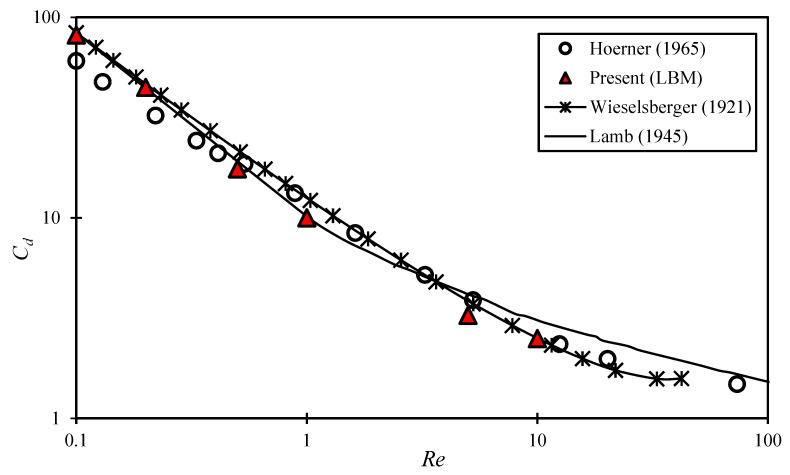
Comparison of current IB-LBM model data with the reported experimental results in refs. [48,49,50].

Finally, the accuracy of the IB-LBM model is confirmed by comparing its results with another famous benchmarking case, which is the fluid flowing around a stationary circular obstacle. The numerical domain with dimensions $L_{x}\times L_{y}$ = 1000×500 is defined, and a circular obstacle with a diameter of 20 units in the computational domain is placed near the inlet. The model is checked for values of Re varying from 0.1 to 10. In each case, the non-dimensional term called ‘Drag coefficient’ $\left(C_{d}\right)$ is calculated using:

(33) $$C_{d}=\frac{F_{drag}}{\frac{1}{2}\rho Au^{r}\left|u^{r}\right|}$$

where $F_{drag}$ represents the drag force per unit length acting on the stationary circular obstacle. It is calculated by vector summing all the forces acting on the membrane nodes in the x-direction. The term ρ is the fluid density, A is the obstacle’s projection area, and $u^{r}$ is the velocity of the obstacle relative to the fluid flow. The numerical results and their validation with the experimental results reported in the literature [48,49,50] are displayed in Figure 5. Again, the numerical results showed an excellent agreement with the experimental results reported in the literature.

## 4. Device Design and Experimental Setup

**Figure 6:**
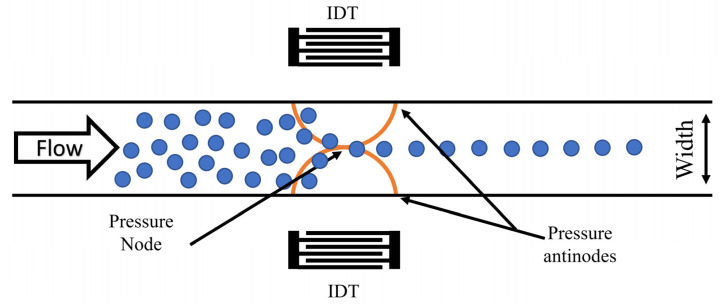
A schematic illustration of the PS particles focusing platform using SSAW. The produced waves consist of two pressure antinodes at the microchannel sidewalls and a single pressure node at the channel’s center.

The SSAW-based focusing platform is a two-layered platform made up of a lithium niobate wafer with two micro interdigitated transducer (IDT) arrays micropatterned on it and bonded to a PDMS microchannel. The platform comprises three primary phases: IDT patterning, PDMS microchannel fabrication, and thermal bonding. This section details the design, microfabrication, and experimental setup employing the above-mentioned microfluidic platform to focus PS particles (diameter = 6.69 µm) at the sidewalls of the microchannel. To accomplish the focusing operation, the channel’s width and height were set at 80 µm, as shown in Figure 6. The electrode pitch was 160 µm while the finger width of each electrode was set to 40 µm. This arrangement generates the required wavelength of 160 µm and frequency of 25 MHz to focus the targeted PS particles. An SSAW is formed when the generated waves from the adjacent IDTs meet inside the microchannel. The produced waves consist of two standing pressure nodes at the microchannel’s sides and a single antinode at the channel’s center. The resonant frequency will cause the PS particles of the positive acoustic contrast factor to migrate toward the pressure nodes [51,52] located at the channel centerline, as illustrated in Figure 6.

The microfabrication of the device starts with patterning IDTs on a lithium niobate (LiNBO_3_) wafer. A chromium layer (thickness = 0.1 µm) is deposited on the LiNBO_3_ wafer via sputtering. Next, the two sets of IDTs are patterned by following all the steps involved in photolithography and wet etching [52]. This involves spin coating a positive photoresist on the LiNBO_3_ wafer and transferring the patterned design to the wafer by exposing the undesired regions of the photoresist to ultraviolet light. The photoresist layer is then developed, removing the exposed areas earlier to the UV light. The residual positive photoresist layers on the unexposed regions mask the underlying chromium layer. The next step involves wet etching, where the unprotected Cr regions are etched away by the metal etchant. Finally, the photoresist layer is stripped off by acetone washing. The second layer of the microdevice includes a microchannel made out of PDMS. A mold is prepared using SU-8 on a silicon (Si) wafer. The PDMS is prepared by mixing the silicone elastomer with the curing agent at a 1:10 ratio. Next, the PDMS is poured onto the prepared mold, degassed in a vacuum chamber, and cured on a hot plate at 80 °C for 2.5 h. Once cooled, the PDMS is peeled off from the mold, and the inlet and outlet holes are punched into it. For the detailed process flow of the microdevice, the reader is referred to our previous publications [53,54].

In the last step, both layers are bonded to finalize the microdevice. Therefore, the lithium niobate wafer containing patterned IDTs on the PDMS microchannel is surface activated in oxygen plasma and immediately attached. Then, the entire device is baked on a hot plate to strengthen the bond between the PDMS channel and the wafer. Finally, both IDT arrays are connected to electric wires using conductive epoxy. Electric wires are used to connect the platform to the power source.

## 5. Results and Discussion

This section mentions the results obtained from the IB-LBM model for a microparticle under SSAW in a continuous-flow manner and their comparison with the experimental results. The main focus is to investigate the motion of microparticles and their behavior when an SSAW is applied to them to focus them on the node location inside the microchannel. However, it should be noted that the physical SSAW force is first scaled properly by following the mapping mechanism in Section 2.2 to correctly apply it in the IB-LBM.

### 5.1. Microparticle Focusing at the Centerline of the Microchannel

The PS particles are taken as the representative particles and deionized (DI) water is the working fluid in the current work. In the first case, the SSAW is applied to the channel in such a manner that the pressure nodes lie in the middle of the channel while the pressure antinodes are located at the channel walls. The simulations are carried out in reduced units in the IB-LBM and are scaled back to the physical units to generate the figures. The wavelength of the applied SSAW is 320 μm. The microchannel’s width is maintained at half the wavelength (i.e., 160 μm) to create a pressure node at the channel’s centerline.

**Figure 7:**
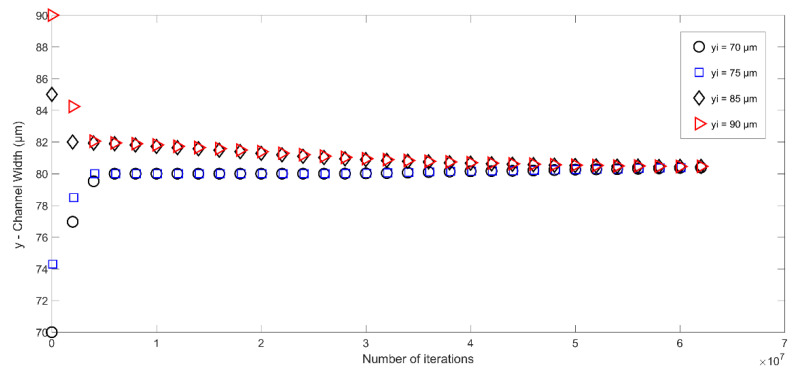
Trajectory of microparticles subjected to acoustic radiation force in microchannel as a function of time and initial position (pressure node is at the center of the microchannel).

Consequently, the acoustic force generated by the applied SSAW drives the PS particles (dia. = 10 μm) toward the middle of the microchannel. The value of Re is kept to ≈0.70 in both numerical and physical domains. Numerous simulations are carried out for different initial positions of the particle in the range of 70 μm to 90 μm; the response of the PS microparticle is reported in Figure 7. Figure 7 demonstrates the trajectory of a microparticle in each case under the effect of surface acoustic waves generated as a result of an applied voltage of 15 $V_{p-p}$. As is evident, the microparticle undergoes two stages during its motion in the microchannel: transient and steady-state stages. The transient state defines the state in which the microchannel is deflected in both x and y directions before it reaches a steady state that defines the pressure node of the acoustic wave. After getting the steady state, the particle’s position changes only in the horizontal direction. In the current investigation, the pressure node is located in the middle of the channel (i.e., 80 µm). Therefore, the particle is settled in its steady state at the centerline of the channel. The particle’s initial position does not matter in this case, and it is deflected toward the pressure node in all cases, irrespective of its initial location at the inlet. This is due to the fact that the opposing forces acting on the particle balance each other at the steady-state locations, and are not affected by the initial locations of the particles.

**Figure 8:**
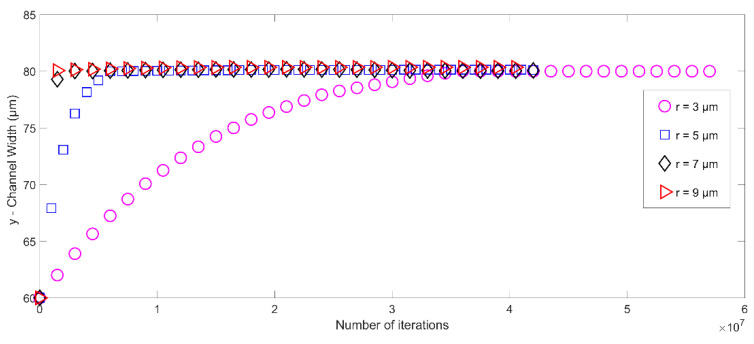
The trajectory of different-sized microparticles subjected to acoustic radiation force in microchannel as a function of time and initial position.

In addition to investigating the impact of the initial location of the microparticle, the model is extended further to study the effect of other geometric parameters on the trajectory of the microparticle in a microchannel. The parameters tested include the microparticle’s radius, density, and compressibility. Figure 8 demonstrates the effect of the microparticle radius on its trajectory under the acoustic radiation force. To this end, the radius of the microparticle is varied from 3 μm to 9 μm while keeping all other properties fixed to compute the motion of the microparticle. The results in Figure 8 depict that the larger particle moves toward the centerline (i.e., pressure node) faster than the smaller particles. This can be explained by the expression of acoustic radiation force, which implies that the acoustic radiation force $\left(F_{A}\right)$ is a function of the cubic radius $\left(r^{3}\right)$ of the particle. Hence, the magnitude of this force is higher for larger particles, causing those particles to move toward the node location faster during their motion in the microchannel. However, the particle’s radius does not affect the final steady-state locations of the target particles at the pressure node as the effective forces (acoustic radiation force and sedimentation force) balance each other at the steady-state location.

**Figure 9:**
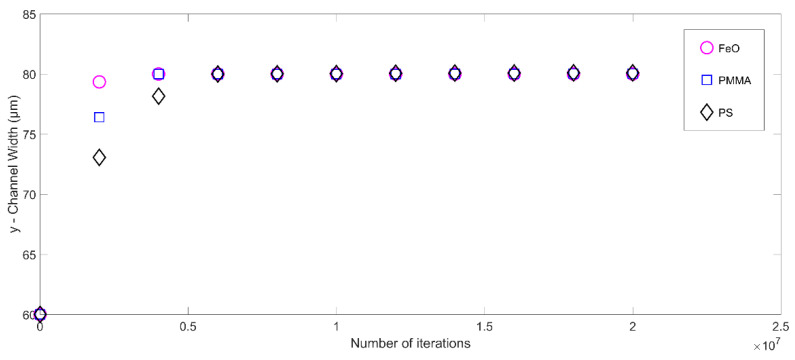
The trajectory of microparticles with different density and compressibility values and subjected to acoustic radiation force in the microchannel.

Next, the numerical model is investigated for three (03) particle types, as represented in Table 2 [55]. The results show that the acoustic radiation force is directly proportional to the particle density, as shown in Figure 9. The particle with a higher density reaches the centerline faster. Since all other particle parameters (such as mass, shape, and size) are kept the same for all particles, the difference in density dictates the transient response of the microparticle and the denser particles have a reduced transient phase. Nevertheless, all the particles ultimately settle at the locations of the pressure nodes. In short, by testing the effect of different parameters, it is clear that varying all these geometric parameters affects the trajectory of the microparticle only in the transient phase. However, the steady-state locations are unaffected by these parameters, as the target particle in all the above cases ultimately settles at the location of the microchannel’s pressure node (here, centerline).

### 5.2. Microparticle Focusing at the Sides of the Microchannel

**Figure 10:**
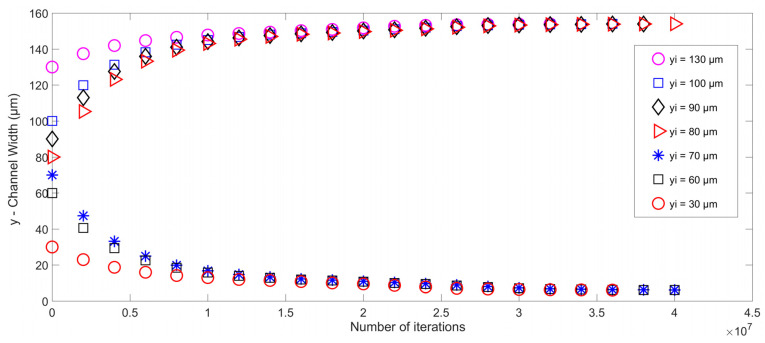
The trajectory of microparticles subjected to acoustic radiation force in the microchannel in the presence of two pressure nodes (at the upper and lower sides) of the microchannel.

As a final case study, the IB-LBM code is modified to have two pressure nodes near the channel walls and a pressure antinode in the middle of the channel. Multiple simulations using PS particles of 6.69 µm diameter are carried out again. Using the same parameters and model discussed previously in this section and changing the position of pressure nodes (from the centerline to the sides of the microchannel), the results are shown in Figure 10, which describes the particle trajectory inside the microchannel. The particles are injected into the microchannel from different initial locations at the inlet. However, as is evident in Figure 10, the change in the initial position of the particle only affects the transient response of the particle. The steady-state locations of the particles remain unchanged as the particle settles at the nearest pressure node location with respect to its initial location in all the cases.

**Figure 11:**
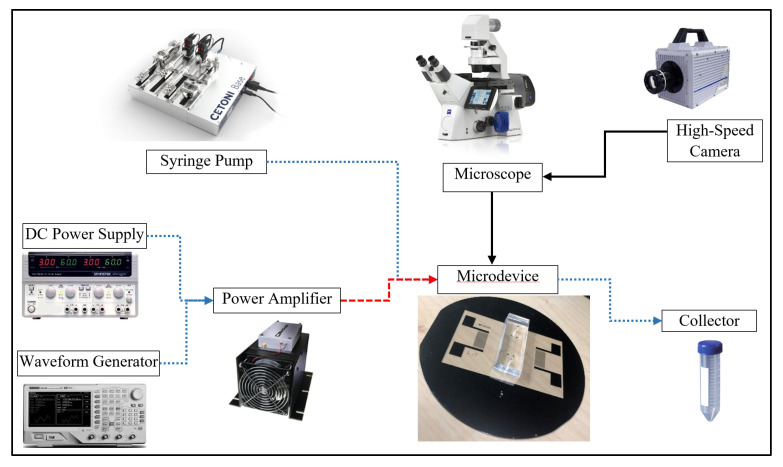
Experimental setup used to perform SSAW-related experiments.

Finally, an experiment to focus the PS particles under the SSAW force is carried out using the fabricated platform to validate the numerical results. The experiments are carried out under an inverted microscope (Zeiss Axio Observer, Carl Zeiss AG, Oberkochen, Germany) connected to a high-speed camera (Fastcam SA-X2, Photron Deutschland GmbH, Reutlingen, Germany) to monitor the response of PS particles. The motion of the particles is recorded in the in-built software, named Photron Fastcam Viewer. The data analysis is then performed using the Photron Fastcam Analysis software (PFA, Photron Deutschland GmbH, Reutlingen, Germany) to measure the displacement, velocity, and acceleration of the target particles. During the experiments, the PS particles of 6.69 µm diameter are suspended in deionized (DI) water (suspension medium) and pumped into the microchannel at a flow rate of 200 µL/h using a syringe pump. A drop of Tween-20 detergent is added to the mixture solution to avoid the adhesion of PS particles to the channel walls. After the fluid flow reached a stable value of 200 µL/h in the microchannel, an AC signal at a frequency of 25 MHz (IDT resonant frequency) and a voltage amplitude of 1 V peak-to-peak is generated using a waveform generator (RIGOL DG4102) and amplified using a power amplifier (ZHL-1-2W-N+) before being applied to the IDT electrodes. The experimental setup is displayed in Figure 11.

**Figure 12:**
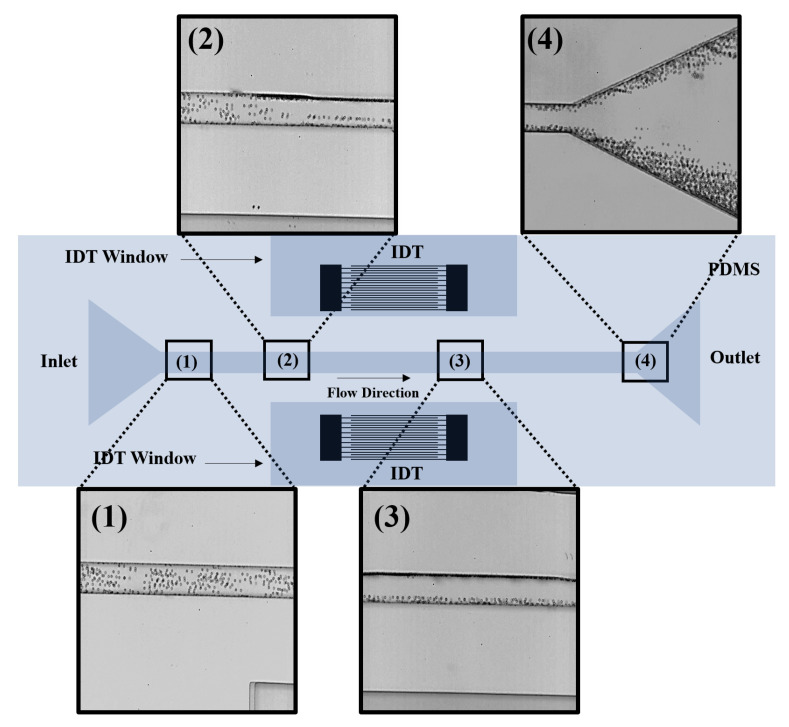
Experimental results of focusing PS particles at pressure nodes under SSAW in the microchannel. (1): microscopic image of PS particles at the inlet section of the platform. PS particles are not yet exposed to SSAW. (2): microscopic image shows the moment PS particles are exposed to SSAW. The exposed PS particles migrate in a lateral direction toward the position of the SAW-created pressure nodes. (3): microscopic image depicts the equilibrium location of PS particles after exposure to SSAW. (4): microscopic image of PS particles at the platform’s outlet after they have been focused.

Figure 12 illustrates the experimental results obtained in the current work. Once the PS particles flow between the two adjacent IDT arrays, they are exposed to acoustic radiation, viscous drag, gravity, and buoyant force. However, the acoustic radiation force is the dominant force on PS particles of a size of 6.69 µm diameter. Figure 12 (1–4) are images of PS particles flowing inside the microchannel captured using the high-speed camera. In Figure 12(1), particles are pumped into the inlet section with no acoustic radiation force effect. The particles are scattered at random within the microchannel. Figure 12(2) shows the stage at which PS particles are affected by the acoustic radiation force. The affected PS particles migrate in a lateral direction toward the position of the SAW-created pressure nodes. Figure 12(3) depicts the equilibrium location of the PS particles after they have exited the section of the channel where they are subjected to acoustic radiation force. Finally, Figure 12(4) shows the effect of SSAW in focusing PS particles. The fabricated platform effectively and efficiently focuses PS particles under SSAW toward the channel walls.

## 6. Conclusions

A numerical model using the IB-LBM is developed to model the motion of a microparticle inside a microchannel subjected to standing surface acoustic waves. All details that are used to represent the fluid, the microparticle and the interaction between them are provided in this paper. It was shown that the trajectory of all the microparticles (having a positive acoustic contrast factor) translates towards the position of the pressure nodes. The particle trajectory’s transient part is affected by several factors, such as the amount of acoustic radiation force, particle radius, density, compressibility, and initial positions of the microparticles. However, the steady-state location of the particles is independent of these parameters and is only a function of the pressure node location inside the microchannel. To support our results and validate our model, a microfluidic device is fabricated in-house, and experiments are designed to mimic the conditions employed in the numerical simulation. An excellent agreement between both results is obtained. By validating the IB-LBM model results with the experiment results, it is shown that the IB-LBM model can accurately model a microparticle’s trajectory in a microchannel accurately. Thus, it is a promising method for designing real LOC devices.

## Figures and Tables

**Table 1:** Parameters used in the current work and their corresponding values in physical and LBM domains.

Property	$\mathrm{Physical}\;\mathrm{Domain}\;\left(phy\right)$	$\mathrm{LBM}\;\mathrm{Domain}\;\left(LBM\right)$	Conversion Factor (C.F.)
Channel width, W	160 μm	80	2 μm
Water density, $\rho_{f}$	1027 kg/m^3^	1	1027 kg/m^3^
Particle density, $\rho_{p}$	1050 kg/m^3^	1.05	1000 kg/m^3^
Fluid Viscosity, ν	1 × 10^−6^ m^2^/s	0.0167	6 × 10^−5^ m^2^/s

**Table 2:** Particles used in the numerical model to study the trajectories with varying density and compressibility values. The properties of the particles are taken from ref [37].

Particle	Particle Density	Particle Compressibility
Polystyrene (PS)	1050 kg/m^3^	2.49 × 10^−10^ pa^−1^
Iron Oxide (FeO)	1500 kg/m^3^	1.5× 10^−11^pa^−1^
Poly(Methyl Methacrylate) (PMMA)	1200 kg/m^3^	1.7 × 10^−10^ pa^−1^

## Data Availability

No new data were created or analyzed in this study.

## References

[1] He S., Joseph N., Feng S., Jellicoe M., Raston C.L. (2020). Application of microfluidic technology in food processing. Food Funct..

[2] Livak-Dahl E., Sinn I., Burns M. (2011). Microfluidic Chemical Analysis Systems. Annu. Rev. Chem. Biomol. Eng..

[3] Wang T., Yu C., Xie X. (2020). Microfluidics for Environmental Applications. Microfluidics in Biotechnology.

[4] Al-Ali A., Waheed W., Abu-Nada E., Alazzam A. (2022). A review of active and passive hybrid systems based on Dielectrophoresis for the manipulation of microparticles. J. Chromatogr. A.

[5] Alhammadi F., Waheed W., El-Khasawneh B., Alazzam A. (2018). Continuous-flow cell dipping and medium exchange in a microdevice using dielectrophoresis. Micromachines.

[6] Sajeesh P., Sen A.K. (2014). Particle separation and sorting in microfluidic devices: A review. Microfluid. Nanofluid..

[7] Zborowski M., Chalmers J.J. (1999). Magnetophoresis: Fundamentals and applications. Wiley Encyclopedia of Electrical and Electronics Engineering.

[8] Destgeer G., Lee K.H., Jung J.H., Alazzam A., Sung H.J. (2013). Continuous separation of particles in a PDMS microfluidic channel via travelling surface acoustic waves (TSAW). Lab A Chip.

[9] Zhang H., Tu E., Hagen N.D., Schnabel C.A., Paliotti M.J., Hoo W.S., Nguyen P.M., Kohrumel J.R., Butler W.F., Chachisvillis M. (2004). Time-of-flight optophoresis analysis of live whole cells in microfluidic channels. Biomed. Microdevices.

[10] Waheed W., Alazzam A., Mathew B., Christoforou N., Abu-Nada E. (2018). Lateral fluid flow fractionation using dielectrophoresis (LFFF-DEP) for size-independent, label-free isolation of circulating tumor cells. J. Chromatogr. B.

[11] Lau A.Y., Hung P.J., Wu A.R., Lee L.P. (2006). Open-access microfluidic patch-clamp array with raised lateral cell trapping sites. Lab A Chip.

[12] Gao Y., Magaud P., Baldas L., Wang Y. (2021). Inertial migration of neutrally buoyant spherical particles in square channels at moderate and high Reynolds numbers. Micromachines.

[13] Soliman A.M., Eldosoky M.A., Taha T.E. (2017). Analysis improvement of standing surface acoustic wave microfluidic devices for bio-particles separation. Int. J. Comput. Appl. Technol..

[14] Lenshof A., Laurell T. (2012). Acoustophoresis. Encyclopedia of Nanotechnology.

[15] Mathew B., Alazzam A., El-Khasawneh B., Maalouf M., Destgeer G., Sung H.J. (2015). Model for tracing the path of microparticles in continuous flow microfluidic devices for 2D focusing via standing acoustic waves. Sep. Purif. Technol..

[16] Nam J., Lee Y., Shin S. (2011). Size-dependent microparticles separation through standing surface acoustic waves. Microfluid. Nanofluid..

[17] Ermak D.L., McCammon J.A. (1978). Brownian dynamics with hydrodynamic interactions. J. Chem. Phys..

[18] Waheed W., Alazzam A., Al-Khateeb A.N., Abu-Nada E. (2020). Dissipative particle dynamics for modeling micro-objects in microfluidics: Application to dielectrophoresis. Biomech. Model. Mechanobiol..

[19] Waheed W., Alazzam A., Al-Khateeb A.N., Sung H.J., Abu-Nada E. (2019). Investigation of DPD transport properties in modeling bioparticle motion under the effect of external forces: Low Reynolds number and high Schmidt scenarios. J. Chem. Phys..

[20] Teschner T.-R., Könözsy L., Jenkins K.W. (2016). Progress in particle-based multiscale and hybrid methods for flow applications. Microfluid. Nanofluid..

[21] Mohamad A. (2011). Lattice Boltzmann Method.

[22] Hardy J., Pomeau Y., De Pazzis O. (1973). Time evolution of a two-dimensional classical lattice system. Phys. Rev. Lett..

[23] Frisch U., Hasslacher B., Pomeau Y. (2019). Lattice-gas automata for the Navier-Stokes equation. Lattice Gas Methods for Partial Differential Equations.

[24] McNamara G.R., Zanetti G. (1988). Use of the Boltzmann equation to simulate lattice-gas automata. Phys. Rev. Lett..

[25] Hsu J.-C., Hsu C.-H., Huang Y.-W. (2019). Acoustophoretic control of microparticle transport using dual-wavelength surface acoustic wave devices. Micromachines.

[26] Gantner A., Hoppe R.H., Köster D., Siebert K., Wixforth A. (2007). Numerical simulation of piezoelectrically agitated surface acoustic waves on microfluidic biochips. Comput. Vis. Sci..

[27] Barnkob R., Augustsson P., Laurell T., Bruus H. (2010). Measuring the local pressure amplitude in microchannel acoustophoresis. Lab A Chip.

[28] Muller P.B., Barnkob R., Jensen M.J.H., Bruus H. (2012). A numerical study of microparticle acoustophoresis driven by acoustic radiation forces and streaming-induced drag forces. Lab A Chip.

[29] Nama N., Barnkob R., Mao Z., Kähler C.J., Costanzo F., Huang T.J. (2015). Numerical study of acoustophoretic motion of particles in a PDMS microchannel driven by surface acoustic waves. Lab A Chip.

[30] Mao Z., Xie Y., Guo F., Ren L., Huang P.-H., Chen Y., Rufo J., Costanzo F., Huang T.J. (2016). Experimental and numerical studies on standing surface acoustic wave microfluidics. Lab A Chip.

[31] Guo F., Mao Z., Chen Y., Xie Z., Lata J.P., Li P., Ren L., Liu J., Yang J., Dao M. (2016). Three-dimensional manipulation of single cells using surface acoustic waves. Proc. Natl. Acad. Sci. USA.

[32] Skov N.R., Bruus H. (2016). Modeling of microdevices for SAW-based acoustophoresis—A study of boundary conditions. Micromachines.

[33] Bach J.S., Bruus H. (2018). Theory of pressure acoustics with viscous boundary layers and streaming in curved elastic cavities. J. Acoust. Soc. Am..

[34] O’Rorke R., Collins D., Ai Y. (2018). A rapid and meshless analytical model of acoustofluidic pressure fields for waveguide design. Biomicrofluid..

[35] Ni Z., Yin C., Xu G., Xie L., Huang J., Liu S., Tu J., Guo X., Zhang D. (2019). Modelling of SAW-PDMS acoustofluidics: Physical fields and particle motions influenced by different descriptions of the PDMS domain. Lab A Chip.

[36] Skov N.R., Sehgal P., Kirby B.J., Bruus H. (2019). Three-dimensional numerical modeling of surface-acoustic-wave devices: Acoustophoresis of micro-and nanoparticles including streaming. Phys. Rev. Appl..

[37] Krüger T., Kusumaatmaja H., Kuzmin A., Shardt O., Silva G., Viggen E.M. (2017). The Lattice Boltzmann method. Springer Int. Publ..

[38] Peskin C.S. (1972). Flow patterns around heart valves: A numerical method. J. Comput. Phys..

[39] Eggleton C.D., Popel A.S. (1998). Large deformation of red blood cell ghosts in a simple shear flow. Phys. Fluids.

[40] Feng Z.-G., Michaelides E.E. (2004). The immersed boundary-Lattice Boltzmann method for solving fluid–particles interaction problems. J. Comput. Phys..

[41] Liu Z., Liu H., Huang D., Zhou L. (2020). The Immersed Boundary-Lattice boltzmann method parallel model for fluid-structure interaction on heterogeneous platforms. Math. Probl. Eng..

[42] Shan X., Chen H. (1993). Lattice Boltzmann model for simulating flows with multiple phases and components. Phys. Rev. E.

[43] Karimnejad S., Delouei A.A., Basagaoglu H., Nazari M., Shahmardan M.M., Falcucci G., Lauricella M., Succi S. (2022). A Review on Contact and Collision Methods for Multi-body Hydrodynamic problems in Complex Flows. arXiv.

[44] Delouei A.A., Nazari M., Kayhani M., Ahmadi G. (2016). A non-Newtonian direct numerical study for stationary and moving objects with various shapes: An immersed boundary–Lattice Boltzmann approach. J. Aerosol Sci..

[45] Munson B., Okiishi T., Huebsch W., Rothmayer A. (2013). Fundamentals of Fluid Mechanics.

[46] Sukop M., Thorne D.T. (2006). Lattice Boltzmann Modeling.

[47] Ghia U., Ghia K.N., Shin C. (1982). High-Re solutions for incompressible flow using the Navier-Stokes equations and a multigrid method. J. Comput. Phys..

[48] White F.M. (1979). Fluid Mechanics.

[49] Hoerner S.F. (1965). Fluid-Dynamic Drag.

[50] Wieselsberger C.v. (1921). Neuere feststellungen uber die gesetze des flussigkeits und luftwiderstands. Phys. Z..

[51] Zhang Y., Chen X. (2021). Particle separation in microfluidics using different modal ultrasonic standing waves. Ultrason. Sonochem..

[52] Mazalan M.B., Noor A.M., Wahab Y., Yahud S., Zaman W.S.W.K. (2021). Current Development in Interdigital Transducer (IDT) Surface Acoustic Wave Devices for Live Cell In Vitro Studies: A Review. Micromachines.

[53] Al-Ali A., Waheed W., Abu-Nada E., Alazzam A. Fabrication of acoustic microfluidic platforms for particle manipulation. Proceedings of the 2020 Advances in Science and Engineering Technology International Conferences (ASET).

[54] Al-Ali A., Waheed W., Abu-Nada E., Mathew B., Sung H.J., Alazzam A. A microfluidic platform with castellated electrodes to separate cancer cells from blood cells. Proceedings of the 2020 International Conference on Manipulation, Automation and Robotics at Small Scales (MARSS).

[55] Simon G., Pailhas Y., Andrade M.A., Reboud J., Marques-Hueso J., Desmulliez M.P., Cooper J.M., Riehle M.O., Bernassau A.L. (2018). Particle separation in surface acoustic wave microfluidic devices using reprogrammable, pseudo-standing waves. Appl. Phys. Lett..

